# α-Synuclein and huntingtin exon 1 amyloid fibrils bind laterally to the cellular membrane

**DOI:** 10.1038/srep19180

**Published:** 2016-01-13

**Authors:** Elodie Monsellier, Luc Bousset, Ronald Melki

**Affiliations:** 1 Paris-Saclay Institute of Neuroscience, Centre National de la Recherche Scientifique, Université Paris-Saclay, Gif-sur-Yvette, France

## Abstract

Fibrillar aggregates involved in neurodegenerative diseases have the ability to spread from one cell to another in a prion-like manner. The underlying molecular mechanisms, in particular the binding mode of the fibrils to cell membranes, are poorly understood. In this work we decipher the modality by which aggregates bind to the cellular membrane, one of the obligatory steps of the propagation cycle. By characterizing the binding properties of aggregates made of α-synuclein or huntingtin exon 1 protein displaying similar composition and structure but different lengths to mammalian cells we demonstrate that in both cases aggregates bind laterally to the cellular membrane, with aggregates extremities displaying little or no role in membrane binding. Lateral binding to artificial liposomes was also observed by transmission electron microscopy. In addition we show that although α-synuclein and huntingtin exon 1 fibrils bind both laterally to the cellular membrane, their mechanisms of interaction differ. Our findings have important implications for the development of future therapeutic tools that aim to block protein aggregates propagation in the brain.

The aggregation of proteins into fibrillar high molecular-weight species is the hallmark of human degenerative diseases, including Alzheimer’s, Parkinson’s, or Huntington’s^1^. During the past decade it has become clear that these fibrillar aggregates have the capacity to spread from one cell to another in a prion-like manner, inducing in the recipient cell the aggregation of like endogenous protein^2^,^3^,^4^,^5^,^6^,^7^,^8^. This unique property could be at the origin of the stereotypic progression of pathology inside the brain^9^. A better knowledge of the molecular and cellular mechanisms underlying the propagation of amyloid fibrils could allow the development of new drugs able to slow down the gradual evolution of the disease.

The docking of extra-cellular aggregates to the plasma membrane of naïve neurons is a key step of the vicious propagation-amplification cycle. Next, the aggregates are internalized and reach the cytoplasm where they can recruit the otherwise soluble like endogenous protein thus elongating/amplifying. The circle completes with the release of the amplified aggregates into the extracellular milieu, where they can target naïve cells. Very little is known about the molecular basis by which protein fibrillar aggregates bind to the plasma membrane. Different mechanisms have been proposed. The binding could be passive^10^, mediated by an interaction with the membrane lipids^11^, and/or with specific protein partners^12^,^13^,^14^. The efficiency of the binding seems to depend both on the aggregates characteristics, such as their charge or conformation^10^,^15^, and on the properties of the membrane, with an emphasis on the role of a specific lipid composition^16^,^17^ or of the membrane curvature^18^,^19^. Whether amyloid fibrils bind to the cellular membrane laterally or through their ends is subject to debate. Indeed, by comparing the permeabilization and toxic properties, which are related to but differ from the binding *per se*, of two types of β-2-microglobulin fibrils displaying different lengths, Xue and Radford proposed that the binding was mediated mainly through fibrils ends^11^,^20^. However massive lateral binding has been also observed in this model like in others^11^,^21^,^22^,^23^. Determining how amyloid fibrils interact with membranes is important owing to the role played by fibril fragmentation, both for the aggregation mechanism^24^,^25^,^26^ and their propagation propensity^20^,^27^. Indeed at any given monomer concentration short, fragmented fibrils display a larger number of extremities as compared to long fibrils while both short and long fibrils have the same lateral surfaces. Thus short fibrils will bind to a higher extent than long fibrils as opposed to with the same efficiency depending on whether interaction with cell membranes occurs through fibrils ends or lateral surfaces.

Here we specifically assessed this issue by studying the binding of amyloid fibrils displaying similar composition and structure but different lengths to artificial liposomes and mammalian cells. We used two different and complementary models, namely α-synuclein (αSyn) involved in Parkinson’s disease, and huntingtin protein exon 1 (HTTExon1) which aggregation is responsible of Huntington’s disease. We observed using electron microscopy massive lateral binding of αSyn and HTTExon1 fibrils to unilamellar vesicles made of brain lipids. We could demonstrate that at identical monomer concentrations, long fibrils made of either αSyn or HTTExon1 bind to the same extent or even better both to artificial liposomes and cells than their short counterparts, thus demonstrating a preeminent role of fibrils surface in the binding. We also show that although αSyn and HTTExon1 fibrils bind both laterally to the cellular membrane, their mechanisms of interaction differ.

## Results

### Preparation of fibrils of different lengths but identical conformation

**Figure 1:**
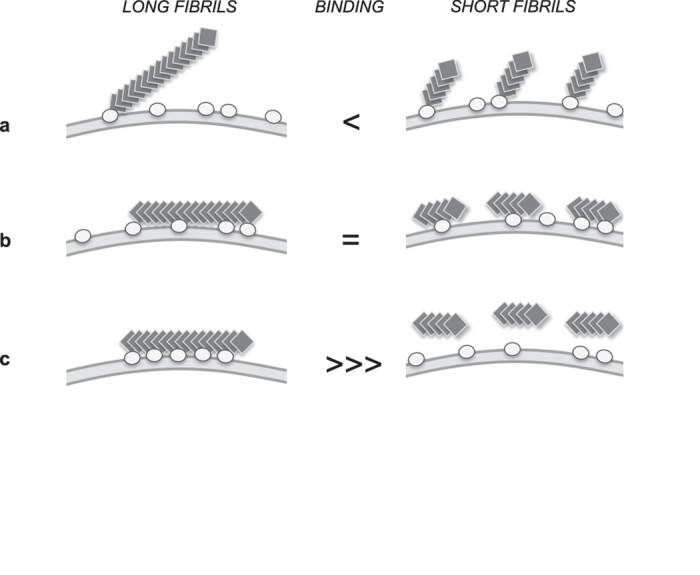
Schematics for the different scenarios of amyloid fibrils binding to biological membranes. (**a**) Binding mainly through fibrils extremities. At identical monomer concentration and under non-saturating conditions, short fibrils will bind the cellular membrane to a higher extent than longer fibrils. (**b**) Lateral binding that does not depend on the number of interacting sites. Long and short fibrils will bind equally well to the cellular membrane. (**c)** Lateral binding with a minimal number of interacting sites required. Binding becomes dependent on fibrils length as the longer the fibrils are the higher the probability of interacting with the minimal number of interaction sites is. Fibrils of a length that do not meet with the required number of interaction sites will not bind the cellular membrane at all.

To determine if amyloid fibrils bind to the cellular membrane laterally or through their extremities, we decided to characterize the binding of fibrils identical in everything but their length (Fig. 1). Consider the case depicted in Fig. 1: at a defined monomer concentration, short fibrils obtained by fragmenting long fibrils are in this example three times shorter than long fibrils; they are therefore three times more concentrated when considering the number of particles and possess three times more extremities but lateral surfaces that are identical in nature and area. We imagined three different scenarios for the binding of these long and short fibrils to the cellular membrane. We reasoned that if fibrils would bind to the cellular membrane exclusively or mainly through their extremities, under non-saturating conditions, short fibrils would bind to a greater extent to the cells than long fibrils, because they have more extremities (Fig. 1a). Conversely, if short fibrils do not bind to a higher extent to cells, then, under non-saturating conditions, fibrils must bind predominantly laterally to the membrane (Fig. 1b,c). Two types of lateral binding may occur: lateral binding that does not depend on the number of binding sites (Fig. 1b), in which case long and short fibrils will have equal binding capacities; and lateral binding that requires a minimal number of binding sites (Fig. 1c). In the later case short fibrils would be too short to simultaneously bind to a sufficient number of anchoring points at the membrane.

**Figure 2:**
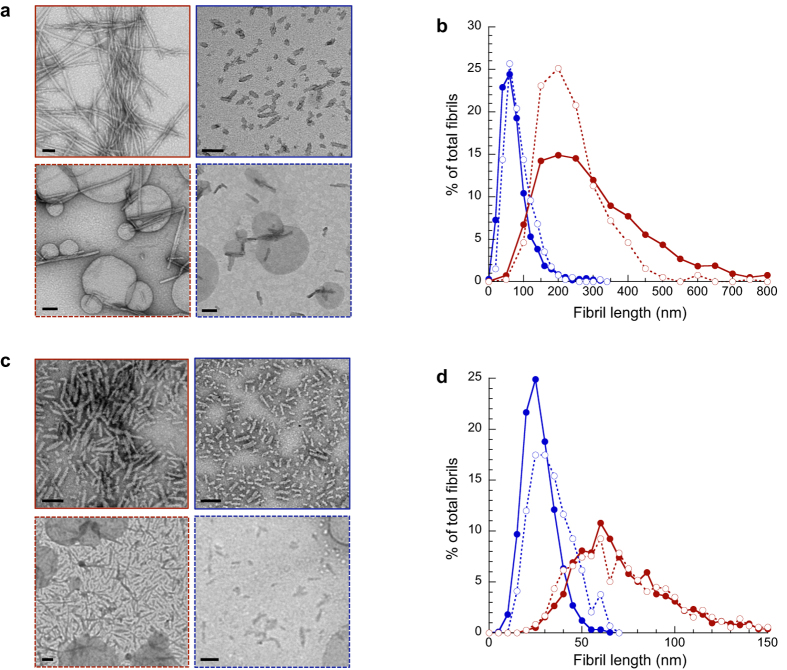
Morphologies and length distributions of long and short αSyn and HTTExon1 fibrils. (**a**,**c**) Representative negatively stained TEM of long (red box) and short (blue box) αSyn (**a**) or HTTExon1 (**c**) fibrils, freshly sonicated (box with solid lines) or after being incubated for 1 hour with unilamellar vesicles made from brain lipids in Dulbecco’s modified Eagle’s medium (box with dashed lines). Scale bar, 100 nm. (**b**,**d**) Length distributions of long (red) and short (blue) αSyn (**b**) or HTTExon1 (**d**) fibrils, freshly sonicated (closed circles, solid lines) or after being incubated for 1 hour with unilamellar vesicles made from brain lipids (open circles, dashed lines). The length distributions were obtained by measuring the length of at least 1000 fibrils from 3 to 10 independent experiments by quantitative negatively stained TEM.

We used sonication to obtain fibrils of different lengths. We assembled αSyn and HTTExon1 into fibrils as described in the Methods section. Each sample was then divided in two: the first one was sonicated for only 10 min, to disperse the aggregates and generate individual long fibrils; the second sample was sonicated for 16 h to break the long fibrils into short ones. Representative images and the distributions of fibril lengths obtained by quantitative electron microscopy for long and short fibrils of each protein are shown in Fig. 2. For both αSyn and HTTExon1 we could obtain two distinct populations of fibrils, displaying similar morphologies but different length distributions.

**Figure 3:**
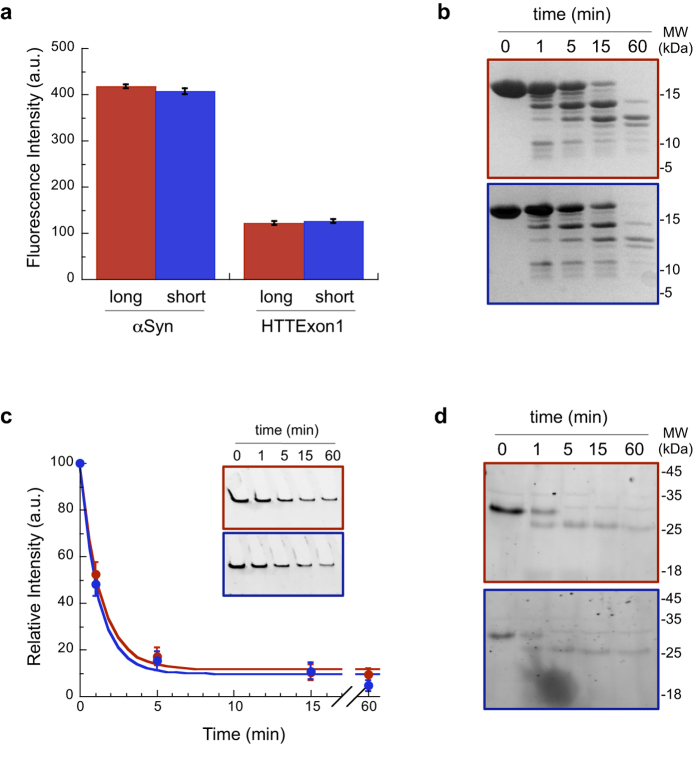
Long and short fibrils have similar structures. (**a**) ThT fluorescence spectra following binding to fibrils. (**b**) Proteinase-K degradation patterns of αSyn long (red) and short (blue) fibrils at identical monomeric protein concentrations. (**c**) Proteinase-K degradation kinetics of SDS-resistant long (red) and short (blue) HTTExon1 fibrils at identical monomeric protein concentrations. Insets, representative SYPRO-stained SDS-PAGE showing the disappearance of fibrillar HTTExon1 trapped within the gel well as a function of time. (**d**) Proteinase-K degradation pattern of TFA-HFIP solubilized long (red) and short (blue) HTTExon1 fibrils as a function of time. SDS-PAGE were SYPRO-stained. In panels a and c means and standard errors are calculated from 3 independent experiments.

Fibrils with different conformations are susceptible to bind differently to the membrane^15^. To check that sonication did not alter fibrils structure we performed several analyses (Fig. 3). Thioflavin T is an amyloid-binding dye which fluorescence intensity depends both on the amount and the conformation of proteins within fibrils^28^. Both αSyn and HTTExon1 long and short fibrils bind Thioflavin T to a similar extent (Fig. 3a). We previously showed that proteinase K degradation patterns can discriminate between different polymorphs of αSyn fibrils^15^. Long and short αSyn fibrils display identical proteinase K degradation patterns (Fig. 3b). This indicates that the protease cleavage sites are distributed similarly in both fibrils. HTTExon1 proteinase K degradation yields fragments that are too short to be resolved by SDS-PAGE, and an SDS-resistant fragment which intensity decreases with time following a kinetics that is similar for long and short fibrils (Fig. 3c). The resolubilisation of this SDS-resistant fragment in TFA:HFIP revealed the progressive disappearance of the band corresponding to full-length HTTExon1 and the progressive appearance of a band with a smaller apparent molecular weight; the pattern and kinetics of appearance and disappearance of these two bands were similar for long and short HTTExon1 fibrils (Fig. 3d).

We conclude from these physico-chemical assessments that we generated αSyn and HTTExon1 fibrils of distinct lengths but identical structures.

### Binding of long and short fibrils to unilamellar vesicles made of brain lipids

**Figure 4:**
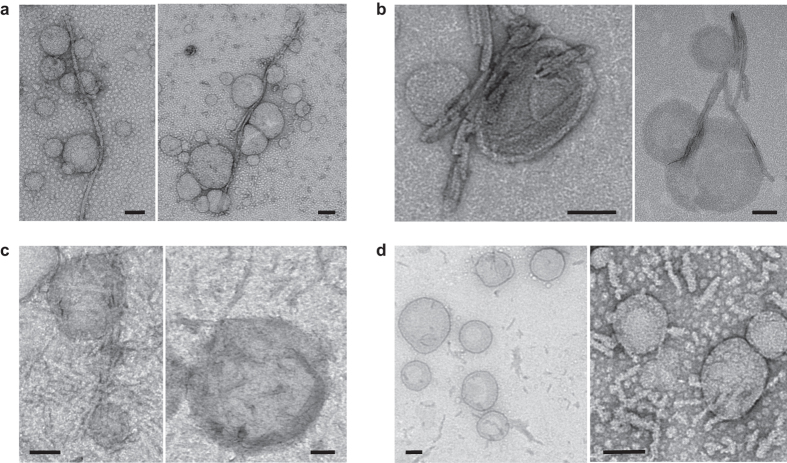
Binding of long and short αSyn and HTTExon1 fibrils to unilamellar vesicles made from brain lipids. Binding of 50 μM αSyn long (**a**) and short (**b**) fibrils and of 50 μM HTTEx1 long (**c**) and short (**d**) fibrils to a 5× mass excess of artificial liposomes made from total brain lipids assessed by TEM. Scale bar, 100 nm.

We used unilamellar vesicles composed of total brain lipids to image the binding of short and long αSyn and HTTExon1 fibrils to lipid membranes. αSyn and HTTExon1 long and short fibrils (50 μM) were incubated for 1 hour at room temperature in Dulbecco’s modified Eagle’s medium containing unilamellar vesicles made of brain lipids. Representative images obtained by electron microscopy are displayed in Fig. 4. As shown in Fig. 4a, liposomes associate to the flank of long αSyn fibrils. The resulting structures look like rosaries, with “beads”, the liposomes, aligned along the “string”, the fibrils. We almost never observed liposomes bound to the end of the fibrils. Short αSyn fibrils also bind to the lipid vesicles (Fig. 4b). As illustrated in Fig. 4b, liposomes bind predominantly laterally to the fibrils. Long HTTExon1 fibrils also bind to liposomes, and as for long and short αSyn fibrils the binding was mostly lateral (Fig. 4c). In a sharp contrast, short HTTExon1 fibrils were almost never associated to liposomes (Fig. 4d). The fibrils being evenly distributed on the grid, the superposition seen between some short HTTExon1 fibrils and liposomes in the 2D-plane of the grid is most probably casual.

We conclude from these experiments that both αSyn and HTTExon1 fibrils bind laterally to artificial liposomes.

### Correlation between fibril length and their cell binding and permeabilization abilities

Artificial liposomes cannot recapitulate the properties of cellular membranes. We therefore assessed the binding of short and long αSyn and HTTExon1 fibrils to cultured cells.

We first controlled that neither the length nor the structure of short and long αSyn and HTTExon1 fibrils were modified upon incubation in a cellular environment. αSyn and HTTExon1 long and short fibrils (50 μM) were incubated in Dulbecco’s modified Eagle’s medium at room temperature with unilamellar vesicles made of brain lipids. Electron microscopy demonstrated that each type of fibrils retain their morphology (Fig. 2a,c) and length upon incubation (Fig. 2b,d). We determined by a filter trap assay followed by western blotting that the amount of fibrillar protein was unchanged (Figure S1a). Finally proteinase K digestion demonstrated that the structure of αSyn (compare Fig. 3b and Figure S1b) or HTTExon1 (compare Fig. 3c and Figure S1c) long and short fibrils was not affected by the experimental conditions (e.g. incubation time, cell growth media and lipid vesicles).

Next we compared the affinity of long and short αSyn and HTTExon1 fibrils for the cellular membrane. αSyn and HTTExon1 fibrils were labelled with Atto 488 before sonication, to ensure that long and short fibrils have identical monomer concentration and fluorescence intensity. We controlled that the labelling altered neither the morphology nor the lengths of the fibrils.

**Figure 5:**
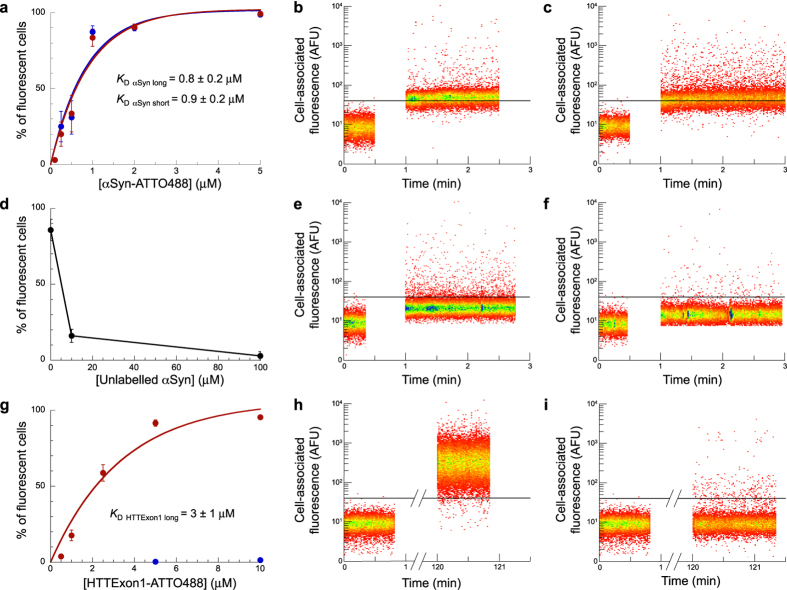
Binding of long and short αSyn and HTTExon1 fibrils to Neuro 2A cells. (**a**) Binding of αSyn-ATTO488 long (red) and short (blue) fibrils at increasing concentrations (0.1–5 μM) to Neuro 2A cells assessed by flow cytometry. (**b**,**c**) Representative traces of the binding of 1 μM αSyn-ATTO488 long (**b**) and short (**c**) fibrils to Neuro 2A cells. (**d**) Binding of 1 μM αSyn-ATTO488 long fibrils to Neuro 2A cells in the presence of increasing concentrations (0–100 μM) of unlabelled long αSyn fibrils. (**e**,**f**) Representative traces of the binding to Neuro 2A cells of αSyn-ATTO488 long fibrils (1 μM) in the presence of 10 (**e**) or 100 (**f**) μM unlabelled long αSyn fibrils. (**g**) Binding of HTTExon1-ATTO488 long and short fibrils at increasing concentrations (0.5–10 μM) to Neuro 2A cells. (**h**,**i**) Representative traces of the binding to Neuro 2A cells of HTTExon1-ATTO488 long fibrils (**h**, 5 μM), and HTTExon1-ATTO488 short fibrils (**i**, 5 μM). The means percentage of cells with bound fibrils and the associated standard error values, calculated from 3 to 6 independent experiments, are represented in (**a**,**d**,**g**). The horizontal line represents the limit between non fluorescent and fibrils-bound, fluorescent cells in (**b**,**c**,**e**,**f**,**h**,**i**).

**Figure 6:**
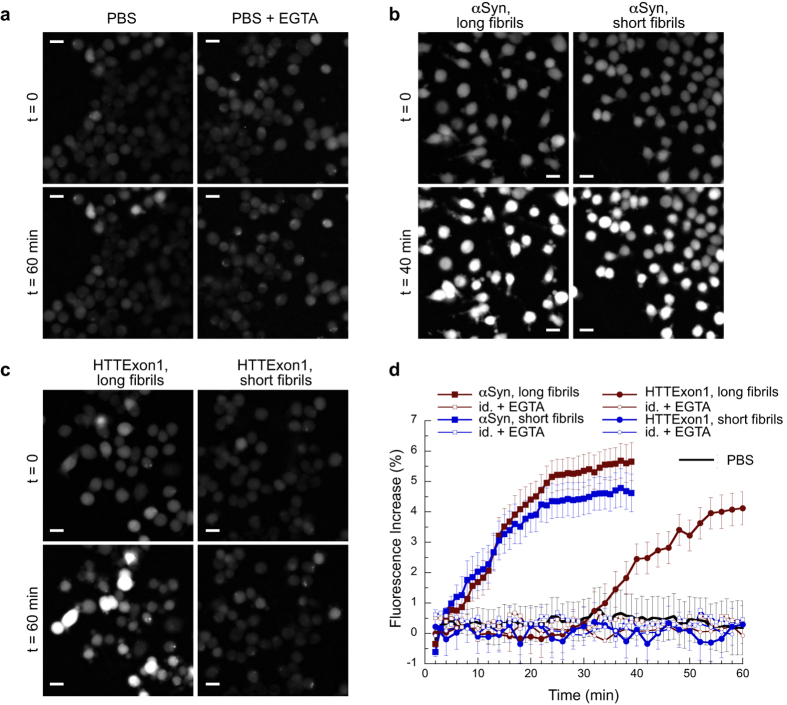
Assessment of Neuro 2A cells permeabilization by long and short fibrils by imaging intracellular free Ca^2+^ alterations. (**a**–**c**) Cells loaded with Fluo4-AM were imaged by epifluorescence microscopy after exposure to PBS or PBS + 5 mM EGTA (**a**), long and short αSyn fibrils (10 μM; **b**), and long and short HTTExon1 fibrils (10 μM; **c**). Scale bars, 20 μm. (**d**) Quantification of the intracellular free Ca^2+^ increase over time, expressed as a fraction of the maximal Ca^2+^ increase observed after addition of ionomycin (10 μM) to the cells. Data are means and associated standard error values calculated from 6 to 10 independent experiments. For the sake of clarity the standard errors are not represented for αSyn and HTTExon1 long and short fibrils in the presence of 5 mM EGTA. For each experiment, fluorescence increase is a mean calculated over ≈50–100 individual cells. The increase in fluorescence measured after addition of PBS + 5 mM EGTA was subtracted for each trace.

As demonstrated previously^15^,^29^, the long αSyn fibrils bound rapidly and efficiently to the plasma membrane of Neuro 2A cells (Figs 5a,b and S2), as assessed by flow cytometry. We measured an observed *K*_D_ for the binding of long αSyn fibrils to Neuro 2A cells of 0.8 ± 0.2 μM (Fig. 5a). We demonstrated that the binding to the cellular membrane was not affected by labelling the protein. Indeed, unlabelled αSyn fibrils competed in a dose-dependent way with the binding of labelled αSyn fibrils to Neuro 2A cells (Fig. 5d–f). The binding of long αSyn fibrils induced a progressive increase in the intracellular free Ca^2+^ level over time as assessed by the increase in the fluorescence of Neuro 2A cells loaded with Fluo-4-AM (Fig. 6a,b,d), similarly to what we reported in earlier studies^15^,^29^. This increase in intracellular free Ca^2+^ level was not due to Ca^2+^ release from intracellular compartment, as no increase in Ca^2+^ level was recorded for cells loaded with Fluo-4-AM upon binding of long αSyn fibrils in the presence of 5 mM EGTA (Fig. 6d). We conclude from these experiments that αSyn long fibrils bind efficiently to the cellular membrane and subsequently induce a permeabilization of the membrane. Remarkably, the binding of short αSyn fibrils was almost identical to that of long fibrils (Figs 5a,c and S2), both under non-saturating and saturating concentrations. We measured an observed *K*_D_ for the binding of short αSyn fibrils to Neuro 2A cells of 0.9 ± 0.2 μM (Fig. 5a), similar to that we determined for long fibrils. In perfect agreement with binding, short αSyn fibrils led to an increase in the intracellular free Ca^2+^ level over time in Neuro 2A cells to a similar extent than long αSyn fibrils (Fig. 6a,b,d). In a manner similar to what we observed for long fibrils, no increase in the intracellular free Ca^2+^ level was recorded when the measurements were performed in the presence of EGTA (Fig. 6d). These observations suggest that the cellular cytoplasmic membrane integrity is compromised upon binding to short αSyn fibrils.

We observed significant differences with fibrillar HTTExon1. As demonstrated previously^10^,^30^ the long HTTExon1 fibrils bound efficiently to the plasma membrane of Neuro 2A cells (Figs 5g,h and S3). We measured an observed *K*_D_ for the binding of long HTTExon1 fibrils to Neuro 2A cells of 3 ± 1 μM (Fig. 5g). This binding was accompanied by a progressive permeabilization of the cellular membrane causing an increase in the intracellular free Ca^2+^ concentration over time (Fig. 6a,c,d), in agreement with previous results^29^. However and contrarily to what was seen with αSyn, we found that even at concentrations that are saturating for the binding of long HTTExon1 fibrils, short HTTExon1 fibrils were neither able to bind to the cellular membrane (Figs 5g,i and S3), nor to permeabilize it (Fig. 6a,c,d).

We conclude from these observations that neither αSyn nor HTTExon1 fibrils bind to cells principally through their ends as otherwise increased binding to cells of short fibrils as compared to long fibrils would have been observed. The short and long αSyn fibrils bind to the membrane with similar affinities and permeabilize it to the same extent. This indicates that αSyn fibrils bind predominantly laterally to the cellular membrane (Fig. 1b). When HTTExon1 fibrils were fragmented by sonication, binding was lost. This cannot be accounted for by fibrils binding through their ends. We therefore conclude that HTTExon1 fibrils also bind laterally to the cellular membrane. However the lateral binding of αSyn and HTTExon1 fibrils differs. While the binding of αSyn to the cellular membrane appears not to depend tightly on the number of interaction sites (Fig. 1b), that of HTTExon1 does given that binding is lost when the fibrils are fragmented. Thus, HTTExon1 fibrils binding appear to depend on a minimal number of interaction sites, a requirement that is fulfilled for long fibrils but not for short ones (Fig. 1c).

## Discussion

It is now well established that fibrillar protein aggregates involved in neurodegenerative diseases spread from one neuron to the other^2^,^3^,^4^,^5^,^6^,^7^,^8^. This propagation cycle comprises different key steps the first of which being the binding of the aggregates to the cellular membrane, followed by their internalization, the recruitment of the endogenous and otherwise soluble cellular proteins into newly born aggregates, and the release of the protein aggregates. Little is known about the molecular mechanisms underlying each of these steps. It has also appeared difficult to study each step independently, many studies focusing on the global cytotoxicity resulting from aggregates internalization^16^,^20^,^27^,^31^. The binding step can be studied *in vitro* using artificial liposomes^11^,^22^,^32^,^33^,^34^, but this strategy neglects the prominent role of membrane proteins and the extracellular matrix in fibrils binding^10^,^13^,^17^,^35^. In this work we demonstrate, by measuring the binding of fibrils displaying identical structures and physico-chemical properties but different lengths to lipid vesicles and cultured cells, that fibril ends are not a prime determinant of the binding process of αSyn and HTTExon1 assemblies.

Lateral binding of fibrils to artificial liposomes was observed by electron microscopy in other models^21^,^22^,^23^. These results together with those we report here using artificial liposomes and cells are different from that obtained with β-2-microglobulin fibrils, which were shown to bind and permeabilize membranes mainly through their ends, although lateral binding was also observed^11^,^20^. While our results do not exclude that αSyn and HTTExon1 fibrils also bind to the cellular membrane through their extremities, they do demonstrate that fibrils ends have an affinity for the membranes that is at most similar to fibrils flanks.

Even if we demonstrate that αSyn and HTTExon1 fibrils bind both laterally to the cellular membrane, the way they interact with the membranes appears to differ significantly. While the binding of αSyn to the cellular membrane seems not to depend on the number of interaction sites (Fig. 1b), that of HTTExon1 does given that binding is lost when the fibrils are fragmented. Thus, HTTExon1 fibrils binding appears to depend on a minimal number of interaction sites, a requirement that is fulfilled for long fibrils but not for short ones (Fig. 1c). We cannot exclude that αSyn fibrils shorter than those we used in this work would exhibit a behaviour similar to that of HTTExon1 short fibrils.

Many fibrils characteristics determine their binding capacities: their net charge^10^, aggregation state^10^,^33^, conformation^15^ or length (this work). Even small sequence modifications can have dramatic effects^10^,^32^,^36^. The next step will be to map fibrils surfaces that binds to the cellular membrane and dissect at the molecular level the binding reaction^37^,^38^,^39^,^40^. The elucidation of this “binding code” is of fundamental importance for the development of new therapeutic tools. For example it has been proposed that fibril stabilization could be valuable to decrease the number of fibrils ends and thus their binding propensity to cells^11^,^24^. While this may impact fibrils uptake because long fibrils could be taken up with lesser efficiency than their short counterparts, our results clearly demonstrate that this strategy is not justified by the somewhat generic ability of amyloid fibrils to bind to cellular membranes through their ends. We therefore favour an alternative approach where fibrils surfaces are modified by fibrils binders that coat their surfaces compromising the crucial step where they interact with cell membranes^41^,^42^.

## Methods

### Expression and purification of αSyn and HTTExon1

Recombinant human wild-type αSyn was expressed in *E. coli* strain BL21(DE3) (Stratagene, La Jolla, CA, USA) and purified as described^43^. Recombinant C-terminally hexa His-tagged MBP-TEV-HTTExon1-His with a polyQ stretch of 48 glutamine residues was expressed in *E. coli* strain BL21(DE3) (Stratagene, La Jolla, CA, USA) and purified as described^44^. HTTExon1-His was obtained from MBP-TEV-HTTExon1-His as described^44^. Briefly, MBP-TEV protease was incubated with the purified MBP-TEV-HTTExon1-His until 100% cleavage was achieved. HTTExon1-His was purified from the resulting mixture using a Talon metal affinity resin column (Clontech, Saint-Germain-en-Laye, France), immediately filtered through a 0.22 μM filter, aliquoted, flash frozen in liquid nitrogen and stored at −80 °C until use.

### Assembly into fibrils, labelling, and preparation of short and long fibrils

For fibril formation, αSyn was incubated in assembly buffer A (50 mM Tris-HCl, pH 7.5, 150 mM KCl) at 37 °C under continuous shaking in an Eppendorf Thermomixer set at 600 rpm; HTTExon1 was incubated in assembly buffer B (20 mM Tris-HCl, pH 7.5, 150 mM KCl, 10% glycerol) at 37 °C without agitation. Long and short fibrils were obtained by sonicating the fibrils with an ultrasond sonicator (Hielsher) set with an amplitude of 75 and a cycle of 0.5 s for 10 min or 16 h, respectively.

For cell sorting experiments, αSyn and HTTExon1 fibrils were first washed in PBS and then labelled by the addition of 2 molar excess of the aminoreactive fluorescent dye Atto 488 N-hydroxysuccinimide ester (ATTO-Tec GmbH) before the sonication step. Labelling was performed following the manufacturer’s recommendation. Unreacted dye was removed by two cycles of sedimentation and suspension of the fibrils in PBS.

### Preparation of unilamellar vesicles made of brain lipids and incubation with fibrils

Porcine brain lipid extract (total) was purchased from Avanti Polar Lipids (Alabaster, AL). Unilamellar vesicles were prepared as previously described^29^ with slight modifications. Briefly, the brain lipids in chloroform were dried in glass tubes under a gentle nitrogen stream. The lipid films were hydrated at 10 mg/ml in Dulbecco’s modified Eagle’s medium, subjected to five freeze-thaw cycles of 2 min each, subjected to sonication for 20 min and centrifuged at 10000 × g for 10 min. αSyn and HTTExon1 long and short fibrils (50 μM) were incubated in Dulbecco’s modified Eagle’s medium for 1 hour at room temperature with unilamellar vesicles at a protein to lipid mass ratio of 1:5.

### Quantitative electronic microscopy

Protein assemblies were examined by transmission electron microscopy (TEM) in a Jeol 1400 transmission electron microscope (Jeol SAS, Croissy-sur-Seine, France) following adsorption onto carbon-coated 200 mesh grids and negative staining with 1% uranyl acetate. The images were recorded with a Gatan Orius CCD camera (Gatan Inc., Pleasanton, CA, USA). Length measurements were performed on individual fibrils with the software ImageJ (NIH, USA). For each fibril type (αSyn long, αSyn short, HTTExon1 long, HTTExon1 short, incubated or not with liposomes) lengths distribution were obtained by measuring the lengths of at least 1000 fibrils, using more than 20 negatively stained electron micrographs obtained from 3 to 10 independent experiments.

### Thioflavin T measurements

αSyn (10 μM monomer concentration) and HTTExon1 (50 μM monomer concentration) long and short fibrils were incubated with Thioflavin T (10 μM). Thioflavin T fluorescence was recorded with a Cary Eclipse spectrofluorimeter (Varian Medical Systems Inc.) using excitation and emission wavelengths set at 440 and 480 nm, respectively.

### Proteolytic digestion and analysis of the proteolytic patterns

αSyn (100 μM monomer concentration) and HTTExon1 (50 μM monomer concentration) long and short fibrils in their respective assembly buffers or incubated in Dulbecco’s modified Eagle’s medium with unilamellar vesicles from brain lipids were treated at 37 °C by Proteinase K (Roche; 3.8 μg.ml^−1^). Aliquots (10 μl) were removed at different time intervals following addition of the protease, immediately mixed with denaturing buffer (10 μl; 180 mM Tris-HCl, pH 6.8, 6% SDS, 15% β-mercaptoethanol, 30% glycerol and 0.01% bromophenol blue) to arrest immediately the cleavage reaction, incubated for exactly 5 min at 90 °C and frozen at −80 °C until analysis on Tris-glycine SDS-polyacrylamide (15%) gel electrophoresis (PAGE). For HTTExon1 aliquots were taken for solubilization. 40 μl of the reaction mixture were withdrawn and immediately diluted in 200 μl of TFA:HFIP 1:1. Fibril solubilization was allowed for 2 hours. The solvent was then removed under a stream of nitrogen for 30 min. After removal from the gas flow the samples were immediately resuspended in 40 μl NaOH 0.1M and then diluted in 40 μl of denaturing buffer. For the analysis of αSyn fibrils cleavage, the gels were stained by coomassie. For the analysis of HTTExon1 fibrils cleavage, the gels were stained with SYPRO Orange (Invitrogen, Paisley, UK) diluted 5000 fold in 10% acetic acid for 1h and visualized using LAS-3000 imager (Fujifilm, Tokyo, Japan).

### Filter trap assay and Western blotting

The amount of long and short aSyn and HTTExon1 fibrils present before and after incubation with unilamellar vesicles from brain lipids in Dulbecco’s modified Eagle’s medium was assessed by a filter retardation assay^45^, where 10 μl of each reaction were withdrawn in triplicate at time zero and after 1 hour of incubation, diluted in 200 μl of sarkosyl 1% (αSyn) or SDS 2% (HTTExon1), filtered through cellulose acetate membranes (0.2 μm pore size, Millipore Corp., Bedford, MA) using a 48-slot slot-blot filtration apparatus (GE Healthcare), and washed twice with 200 μl of sarkosyl 1% (αSyn) or SDS 2% (HTTExon1). The cellulose acetate membranes were incubated with 3% skim milk, probed with a mouse monoclonal anti-αSyn antibody (BD Transduction Laboratories) or a rabbit polyclonal anti-HTTExon1 antibody we raised, and developed with the enzyme-coupled luminescence technique (ECL, Thermo Scientific) following the manufacturer recommendations.

### Cell culture

Murine neuroblastoma Neuro 2a cells (ATCC) were culture at 37 °C in humidified air with 5% CO_2_ in Dulbecco’s modified Eagle’s medium containing 10% foetal bovin serum, 2 mM glutamine, 100 units.ml^−1^ penicillin and 100 μg.ml^−1^ streptomycin. All materials used for cell culture were from PAA Laboratories GmbH (Pasching, Austria).

### Cell sorting experiments

To monitor the binding of αSyn long and short fibrils to the cell membranes, Neuro 2A cells lifted with EDTA were resuspended in serum-free, phenol red free-Dulbecco’s modified Eagle’s medium containing 1 μg.ml^−1^ of propidium iodide. At time zero, Atto 488-labelled αSyn long or short fibrils at increasing monomer concentrations (0.1–5 μM) were added to the cells (10^6^ cells per ml) and analysis were performed using a MoFlo Astrios Cell Sorter (Beckman Coulter). The cell population was gated by forward and side scatter and at least 15,000 cells were recorded. The fraction of fluorescent cells was calculated over a time frame where the signal was stable.

As the binding of HTTExon1 fibrils to cells is a slow process^30^, we proceeded differently. 10^5^ cells were platted in a 6 cm-diameter Petri dish 48 h before the experiment. They were then washed with PBS and incubated with Atto 488-labelled HTTExon1 long or short fibrils at increasing monomer concentrations (0.5–10 μM) for 2 h at 37 °C. After incubation cells were lifted with EDTA and analysed as described above.

### Intracellular Ca^2+^ levels measurements and epifluorescence microscopy imaging

Subconfluent Neuro 2A cells cultured on ibidi-μ-Dishes (Biovalley) were loaded for 30 min at 25 °C with 2 μM of the fluorescent dye Fluo-4-acetoxymethyl ester (Fluo-4-AM) (Invitrogen) in the presence of 0.02% (w/v) Pluronic Acid F127 (Invitrogen) and 2.5 mM of the organic anion-transport inhibitor probenicid (Invitrogen). After washing the cells deesterification of the Fluo-4-AM was allowed for 10 min at 25 °C in serum-free Dulbecco’s modified Eagle’s Medium in the presence of 0.02% (w/v) Pluronic Acid F127 (Invitrogen). PBS, PBS + EGTA (5 mM), αSyn or HTTExon1 long or short fibrils (10 μM monomer concentration) with or without EGTA (5 mM) were then added to the cells. The cells were imaged over time on the Axio Observer ZI epifluorescence microscope (Zeiss) at a magnification of 20x. Images were acquired every 1 (αSyn) or 2 (HTTExon1) min for 40 (αSyn) or 60 (HTTExon1) min at 25 °C in humidified air with 5% CO2. The fluorescence was quantified for each cell of the field (≈50–100 cells/field) using the software ImageJ, after background subtraction for each image, and expressed as a fraction of the maximum fluorescence recorded upon addition of ionomycin (10 μM) to the cells.

## Additional Information

**How to cite this article**: Monsellier, E. *et al.* α-Synuclein and Huntingtin exon 1 amyloid fibrils bind laterally to the cellular membrane. *Sci. Rep.* **6**, 19180; doi: 10.1038/srep19180 (2016).

## Supplementary Material

Supplementary Information
