# Supplementary material for: α-Synuclein and huntingtin exon 1 amyloid fibrils bind laterally to the cellular membrane
Source: Sci Rep. 2016 Jan 13;6:19180. doi: 10.1038/srep19180 (PMC4725933; doi:10.1038/srep19180)
Supplement: Supplementary Information [file srep19180-s1.pdf]

α-Synuclein and huntingtin exon 1 amyloid fibrils bind laterally to the cellular membrane

Elodie Monsellier*, Luc Bousset and Ronald Melki*

Paris-Saclay Institute of Neuroscience, Centre National de la Recherche Scientifique, Université Paris-Saclay, Gif-sur-Yvette, France

** To whom correspondence should be addressed:*
Elodie Monsellier, +33-16982-3486, monsellier@lebs.cnrs-gif.fr
Ronald Melki, +33-16982-3503, melki@lebs.cnrs-gif.fr

SUPPLEMENTARY FIGURES

Supplementary Figure 1. The structure of long and short fibrils is unchanged upon incubation with unilamellar vesicles made from brain lipids in Dulbecco's modified Eagle's medium.
Long and short αSyn and HTTExon1 fibrils (50 mM) were incubated for 1 hour at room temperature with unilamellar vesicles made from brain lipids at a protein to lipid ratio of 1:5 in Dulbecco's modified Eagle's medium. Aliquots were withdrawn at different time intervals and analyzed by filter trap (**a**) or proteinase K digestion (**b,c**). Red boxes, long fibrils; blue boxes, short fibrils; boxes with solid lines, fibrils freshly mixed with brain lipid unilamellar vesicles; boxes with dashed lines, fibrils incubated for 1 hour with brain lipid unilamellar vesicles. The results have to be compared with the ones obtained for fibrils without liposomes (Figure 3).

Supplementary Figures 2 and 3. Binding of long and short αSyn (Supplementary Figure 2) and HTTExon1 (Supplementary Figure 3) fibrils to Neuro 2A cells.
Representative traces of the binding of αSyn-ATTO488 (0.1 – 5 mM) and of HTTExon1-ATTO488 (0.5 – 10 mM) long and short fibrils at increasing concentrations to Neuro 2A cells assessed by flow cytometry. Three independent measurements were performed. The horizontal line represents the limit between non fluorescent and fibrils-bound, fluorescent cells.

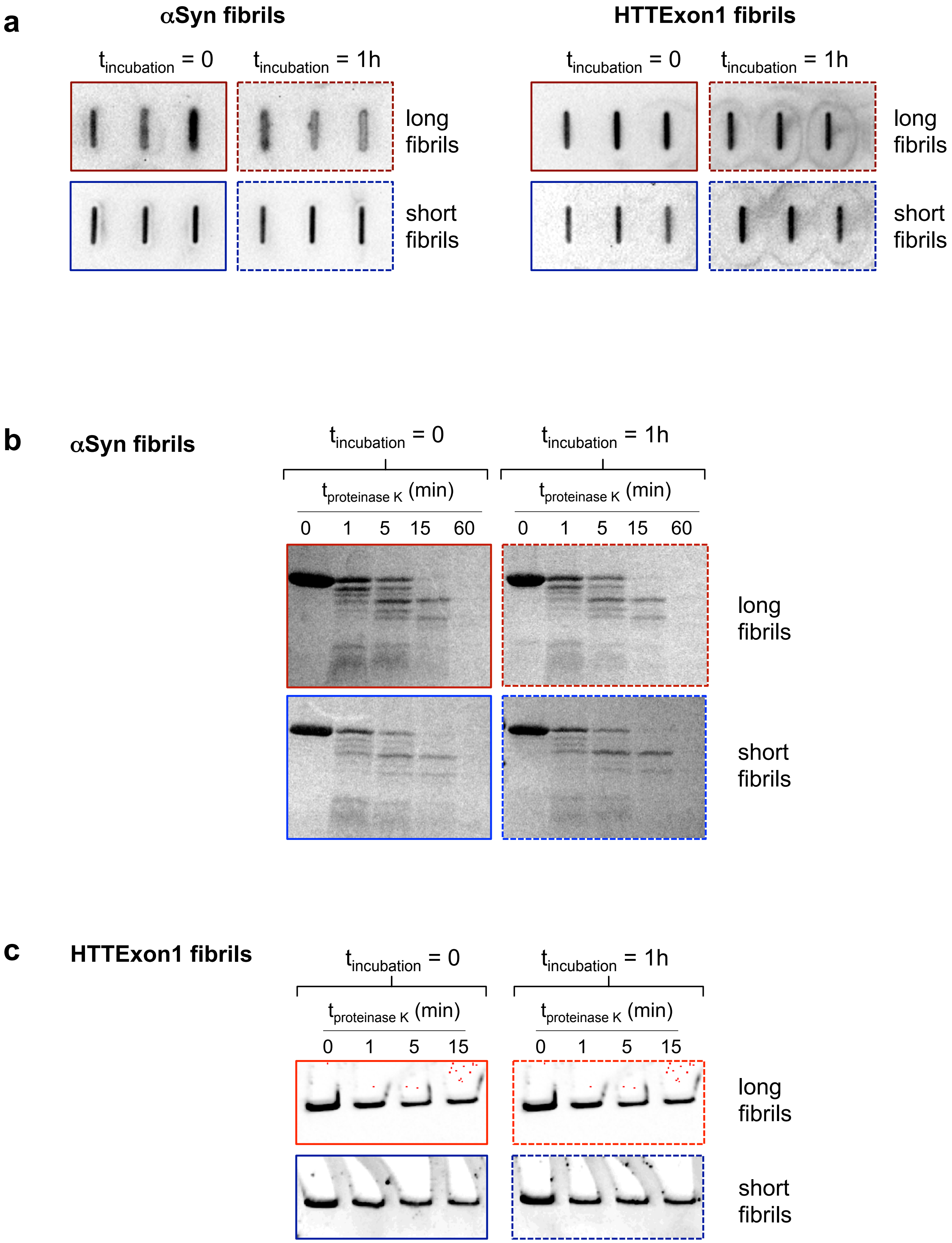

Supplementary Figure 1

αSyn long fibrils:

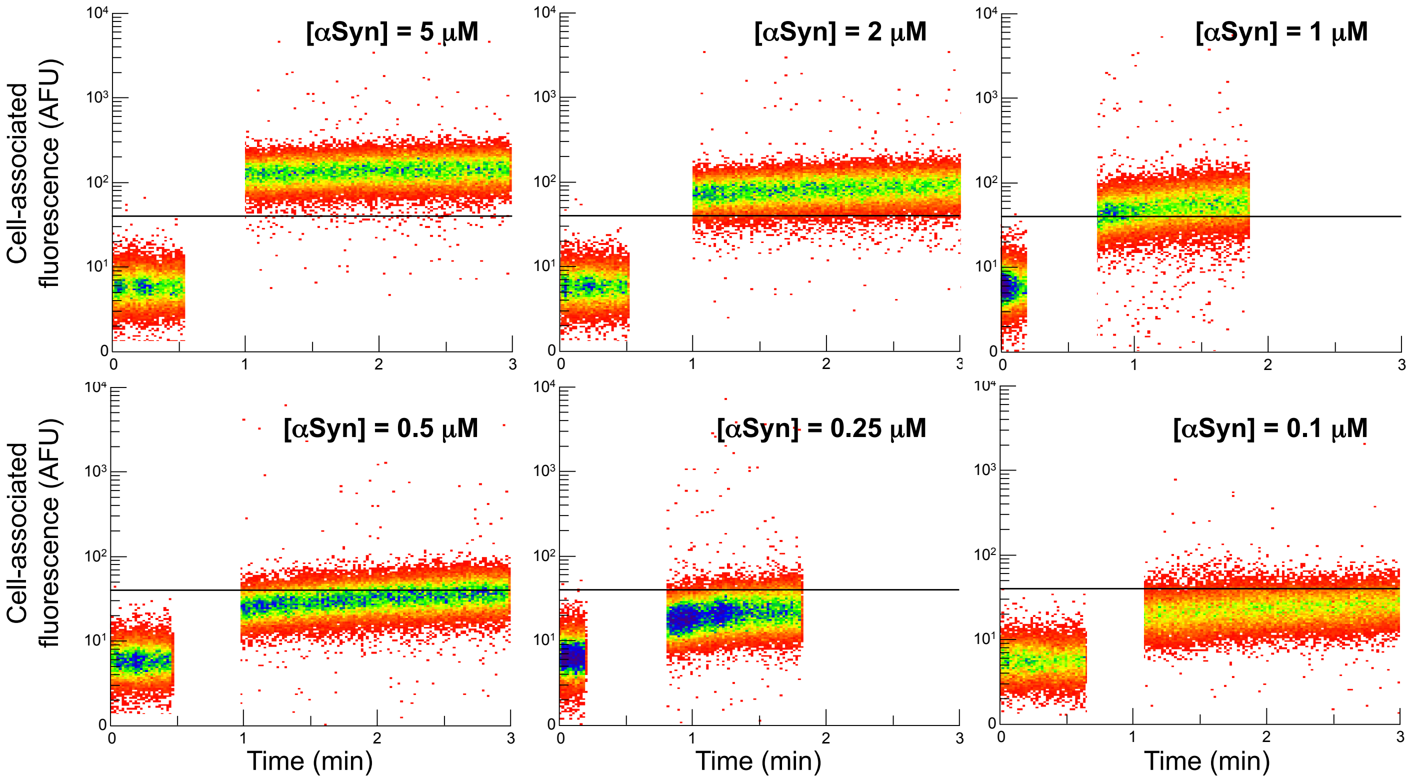

αSyn short fibrils:

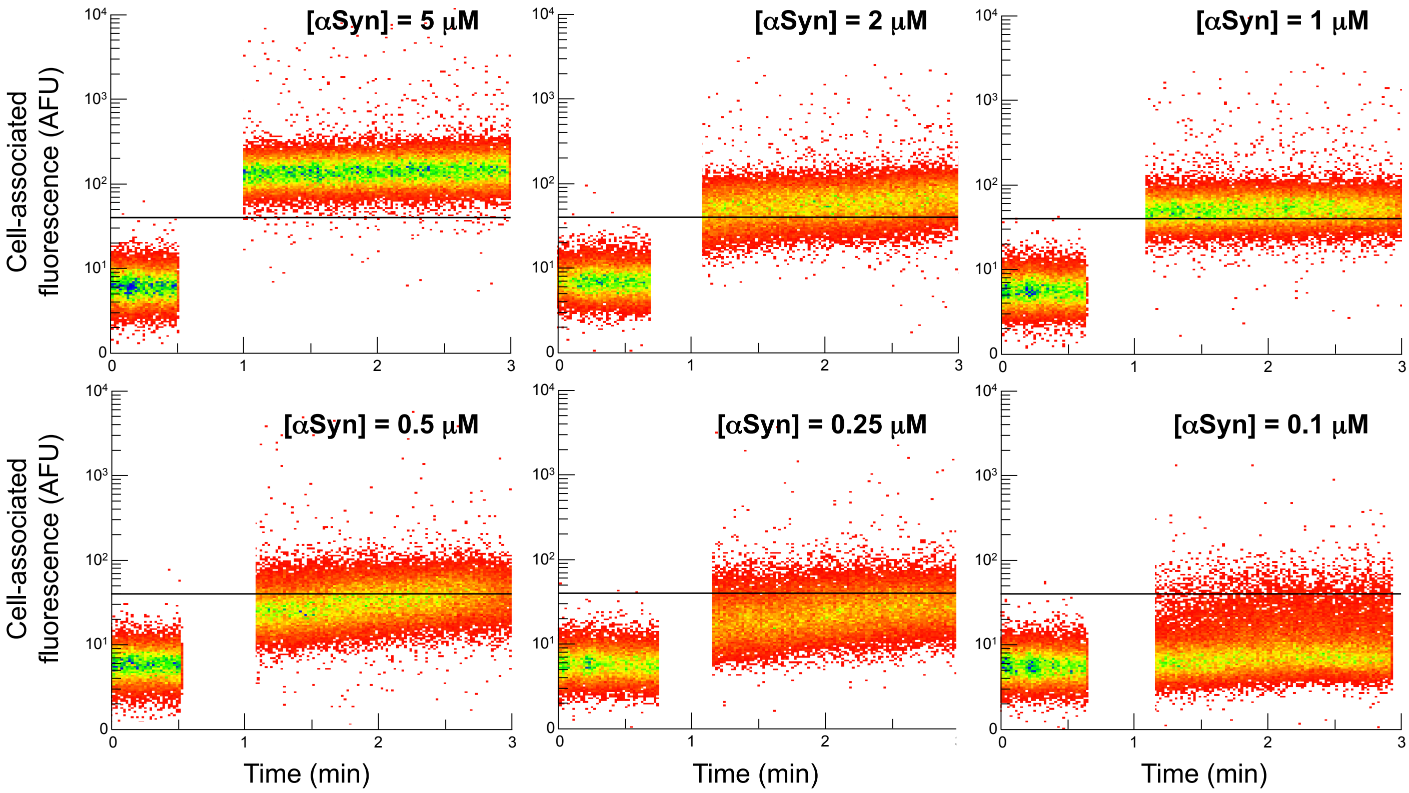

Supplementary Figure 2

HTTExon1 long fibrils:

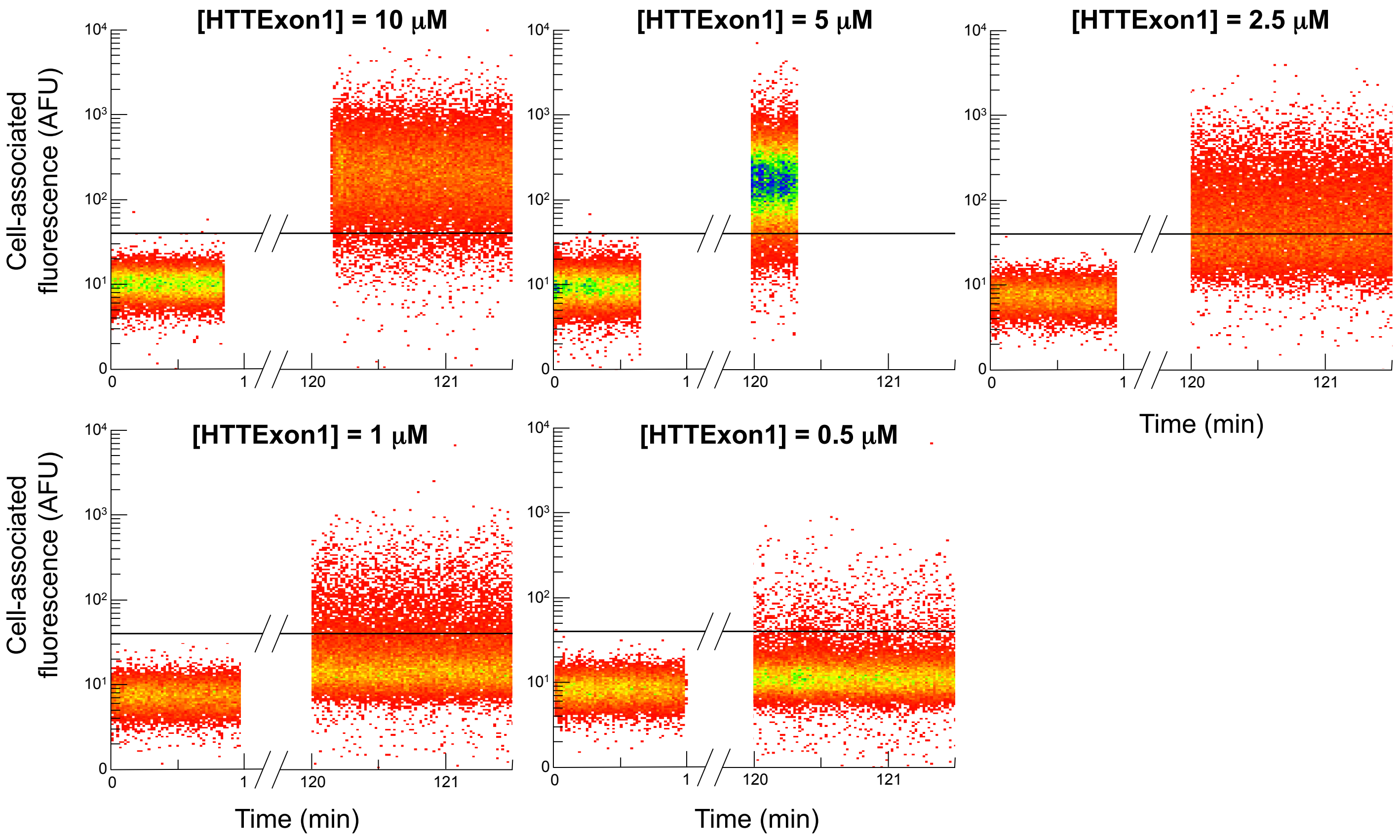

HTTExon1 short fibrils:

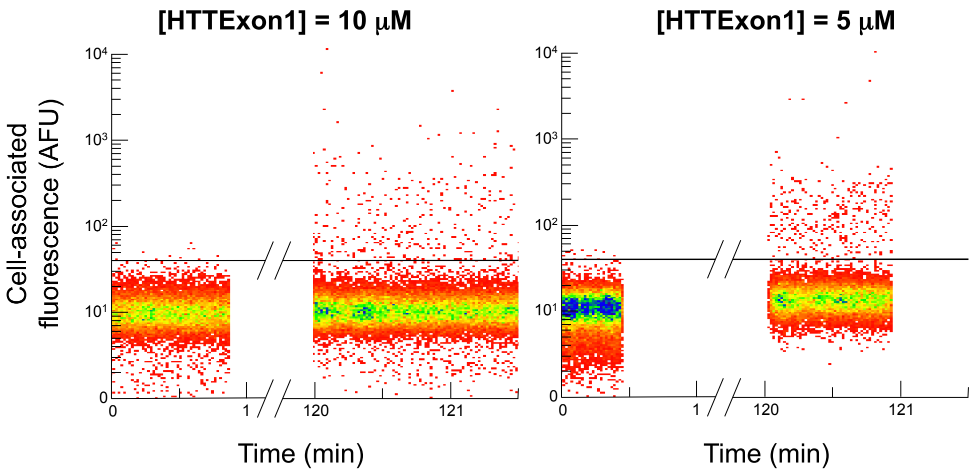

Supplementary Figure 3
